# Antibody Responses to *Mycoplasma pneumoniae*: Role in Pathogenesis and Diagnosis of Encephalitis?

**DOI:** 10.1371/journal.ppat.1003983

**Published:** 2014-06-12

**Authors:** Patrick M. Meyer Sauteur, Bart C. Jacobs, Emiel B. M. Spuesens, Enno Jacobs, David Nadal, Cornelis Vink, Annemarie M. C. van Rossum

**Affiliations:** 1 Department of Pediatrics, Division of Pediatric Infectious Diseases and Immunology, Erasmus MC–Sophia Children's Hospital, University Medical Center, Rotterdam, The Netherlands; 2 Laboratory of Pediatrics, Erasmus MC–Sophia Children's Hospital, University Medical Center, Rotterdam, The Netherlands; 3 Division of Infectious Diseases and Hospital Epidemiology, University Children's Hospital of Zurich, Zurich, Switzerland; 4 Children's Research Center (CRC), University Children's Hospital of Zurich, Zurich, Switzerland; 5 Department of Neurology, Erasmus MC, University Medical Center, Rotterdam, The Netherlands; 6 Department of Immunology, Erasmus MC, University Medical Center, Rotterdam, The Netherlands; 7 TU Dresden, Medical Faculty Carl Gustav Carus, Institute of Medical Microbiology and Hygiene, Dresden, Germany; 8 Erasmus University College, Erasmus University, Rotterdam, The Netherlands; University of Notre Dame, United States of America

The pathogenesis of encephalitis associated with the respiratory pathogen *Mycoplasma pneumoniae* is not well understood. A direct infection of the central nervous system (CNS) and an immune-mediated process have been discussed [1]. Recent observations suggest that intrathecally detectable antibodies against the bacterium, which can serve to establish the etiology of encephalitis, may indeed mediate the disease. 

*Mycoplasma pneumoniae* is a major cause of upper and lower respiratory tract infections in humans worldwide, particularly in children [2], [3]. Up to 40% of community-acquired pneumonia in children admitted to the hospital are attributed to *M. pneumoniae* infection [4]–[7]. Although the infection is rarely fatal, patients of every age can develop severe and fulminant disease. Apart from the respiratory tract infection, *M. pneumoniae* can cause extrapulmonary manifestations. They occur in up to 25% of manifest *M. pneumoniae* infections and may affect almost every organ, including the skin as well as the hematologic, cardiovascular, musculoskeletal, and nervous system [8]. Encephalitis is one of the most common and severe complications [1]. *M. pneumoniae* infection is established in 5%–10% of pediatric encephalitis patients [9], [10], and up to 60% of them show neurologic sequelae [10], [11].

It is important to establish the cause of encephalitis at an early stage in order to specifically treat what can be treated and to avoid unnecessary treatment. The diagnosis of *M. pneumoniae* encephalitis is challenging. The current diagnostic algorithm of the “Consensus Statement of the International Encephalitis Consortium” [12] recommends for the diagnosis of *M. pneumoniae* infection in children with encephalitis (1) serology and polymerase chain reaction (PCR) from throat samples (routine studies), and if positive test results and/or respiratory symptoms are present, then (2) additionally PCR in cerebrospinal fluid (CSF) (conditional studies).

However, *M. pneumoniae* serology and PCR in the respiratory tract cannot discern between colonization and infection in a clinically relevant time frame [13]. The main reason for this is the relatively high prevalence of *M. pneumoniae* in the upper respiratory tract of healthy children (up to 56%) [13], [14]. The demonstrated positive serological results in such asymptomatic PCR-positive children (positive immunoglobulin (Ig) M in 17%, IgG in 24%, and IgA in 6% of 66 cases) [13] may simply reflect one or more previous encounters with *M. pneumoniae* and are not necessarily related to the presence of *M. pneumoniae* in the respiratory tract. It is clear that this may give rise to an overestimation of the *M. pneumoniae*-related disease burden. A more reliable diagnosis of *M. pneumoniae* infection may be achieved by using paired patient sera in order to detect seroconversion and/or a 4-fold increase in antibody titers in addition to PCR (Table 1; table references: [13], [15]–[24]). However, such procedures are time-consuming and are therefore neither practicable nor useful in an acutely ill patient.

**Table 1:** Overview of diagnostic tests for *M. pneumoniae*.

Method	Test	Target/Antigen	Antibodies	Specimen	Performance^1^	Value	Comments
Direct identification of *M. pneumoniae*	PCR	Different target genes (e.g., P1 gene, 16S rDNA, 16S rRNA, RepMP elements, etc.)	-	Respiratory specimen (nasopharyngeal secretion, pharyngeal swab, sputum, bronchoalveolar lavage), CSF, and other bodily fluids or tissues	High sensitivity, high specificity	RD^2^	NAATs provide fast results (in less than a day) and may be earlier than serology (because antibody production requires several days); validation and standardization required for routine diagnostic
	Culture	-	-	Respiratory specimen (see above)	Low sensitivity, high specificity	AD	Special enriched broth or agar media; isolation takes up to 21 days
Nonspecific serological tests for *M. pneumoniae*	Cold-agglutinin test (“bedside test”)	Erythrocytes (I antigen)	Cold agglutinins (IgM)	Serum	Low sensitivity, low specificity	-3	Cold agglutinins target the I antigen of erythrocytes (alternative theory: cold agglutinins target directly *M. pneumoniae* adhered to erythrocytes); positive in only about 50% and in the first week of symptoms; less well studied in children; cross-reactivity with other pathogens and noninfectious diseases
Specific serological tests for *M. pneumoniae*	CFT	Crude antigen extract with glycolipids and/or proteins	Igs (no discrimination between isotypes)	Serum	Sensitivity and specificity comparable to EIA	-3	Positive criteria: 4-fold titer increase between acute and convalescent sera or single titer ≥1∶32; cross-reactivity with other pathogens and noninfectious diseases
	PA		IgM and IgG simultaneously			-3	See above
	EIA	Proteins (e.g., adhesion protein P1) and/or glycolipids	IgM, IgG,^4^^,^ 5 (IgA)^6^	Serum^4^, CSF^5^^,^ 7, other bodily fluids^7^	Moderate-high sensitivity, moderate-high specificity	RD	The sensitivity depends on the time point of the first serum and on the availability of paired sera (for seroconversion and/or rise in titer); “gold standard”: 4-fold titer increase as measured in paired sera
	Immunoblotting				High sensitivity, high specificity^8^	AD	Confirmatory assay
	IFA				Less sensitive and less specific than EIA	AD	Subjective interpretation

Abbreviations: AD, advanced diagnostic test; CFT, complement fixation test; CNS, central nervous system; CSF, cerebrospinal fluid; EIA, enzyme immunoassay; IFA, immunofluorescent assay; Ig, immunoglobulin; NAATs, nucleic acid amplification tests; PA, particle agglutination assay; PCR, polymerase chain reaction; RD, routine diagnostic test; RepMP, repeated *M. pneumoniae* DNA. References: [13], [15]–[24].

1 Qualitative statements included because of the wide range of test performances, which depend on the assay, the patient cohort (children and/or adults), the reference standard (PCR, culture, and/or serology), the respiratory specimen (for PCR), and the time point of the sample collection after disease onset (for EIA)—e.g., sensitivities and specificities for PCR [17], [18]: 79%–100% and 96%–99%; IgM EIA (in relation to PCR) [19]: 35%–77% and 49%–100%; and for IgG EIA [17], [19]: 37%–100% (no indication on specificity because of missing information on previous *M. pneumoniae* infections).

2 Epidemiological differentiation of clinical strains on the basis of differences in the P1 gene by PCR or in the number of repetitive sequences at a given genomic locus by multilocus variable-number tandem-repeat analysis (MLVA) [23].

3 Largely replaced by EIA.

4 **Kinetics of antibody responses in blood.** IgM: onset: within 1 week after the onset of symptoms; peak: 3–6 weeks; persistence: months (to years). IgG: onset and peak: 2 weeks after IgM; persistence: years (to lifelong); reinfection in adults may lead directly to an IgG response in the absence of an IgM response. IgA: onset, peak, and decrease earlier than IgM.

5 **Antibody responses in the CNS differ from blood.** There is no switch from an IgM to an IgG response, the pattern of IgM, IgG, and IgA synthesis remains rather constant and depends on the cause, and there is a long-lasting and slow decay of intrathecal antibody synthesis [22]. In *M. pneumoniae* encephalitis, a dominant IgM response has been observed [29].

6 The prevalence of serum IgA determined by EIA has been shown to be very low in PCR-positive children with symptomatic respiratory tract infection (2.0%) [13].

7 To our knowledge, no validated test is available.

8 Immunoblotting with a combination of at least five specific *M. pneumoniae* proteins showed sensitivities (in relation to PCR) of 83% (IgM), 51% (IgG), and 64% (IgA), and specificities of 94%–100% (IgM), 98%–100% (IgG), and 93%–97% (IgA) [24].

**Figure 1:**
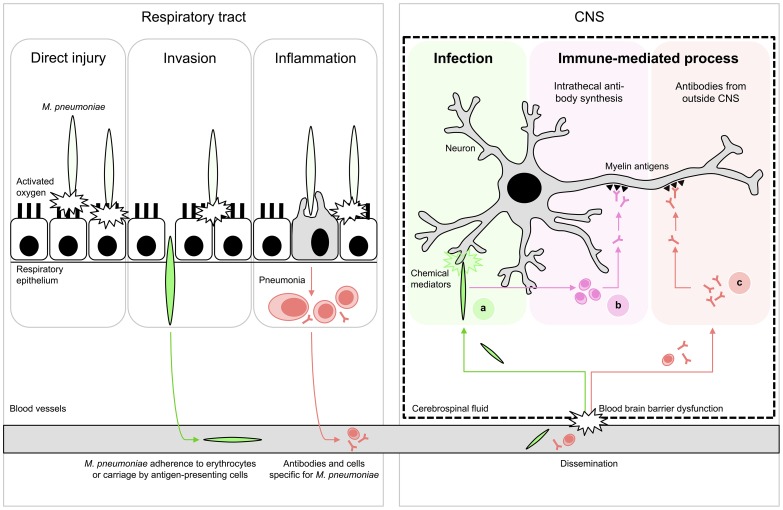
Proposed schematic pathomechanisms in *M. pneumoniae* encephalitis. (Left) Respiratory tract infection. *M. pneumoniae* resides mostly extracellularly on epithelial surfaces. Its close association allows the production of direct injury by a variety of local cytotoxic effects. Furthermore, it can induce inflammatory responses, elicited by both adhesion proteins and glycolipid epitopes that result in pneumonia. (Right) Encephalitis. Extrapulmonary disease of the CNS is characterized by systemic dissemination with resultant direct infection and local tissue injury (A) or immune-mediated injury (B,C). The latter may occur as a result of cross-reactive antibodies against myelin components, e.g., gangliosides and galactocerebroside C. These antibodies could theoretically have originated from intrathecal synthesis (B) or from outside the CNS (C). Figure adapted from [1]; see references in the text.

The detection rate of *M. pneumoniae* by PCR in the CSF of *M. pneumoniae* encephalitis patients is relatively low (0%–14%) [9], [10], [25], [26]. Moreover, various cases with *M. pneumoniae* encephalitis in which bacterial DNA could not be detected in the CSF had a more prolonged duration of respiratory symptoms before the onset of encephalitis (>5–7 days) [10], [25], [27]. These cases indicate that *M. pneumoniae* encephalitis may exemplify a postinfectious phenomenon that manifests after clearance of the bacteria from the CNS or respiratory tract by the immune system. The immune response to *M. pneumoniae* in the CNS or other sites may also contribute to the encephalitis (Figure 1; figure references: [1]).

**Figure 2:**
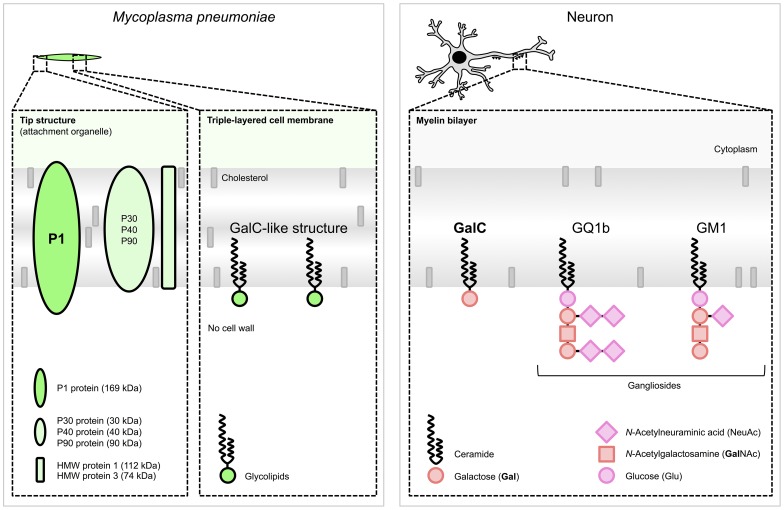
Schematic structures responsible for molecular mimicry between *M. pneumoniae* and neuronal cells. (Left) *M. pneumoniae* adhesion proteins and glycolipids. The immunogenic and major cytadherence proteins P1 and P30 are densely clustered at the tip structure. The P1 protein [31] and glycolipids, e.g., those forming a GalC-like structure [32], elicit cross-reactive antibodies induced by molecular mimicry. (Right) Host myelin glycolipids, to which antibodies were found in patients with *M. pneumoniae* encephalitis. Glycolipids are organized in specialized functional microdomains called “lipid rafts” and play a part in the maintenance of the cell membrane structure. Abbreviations: GalC, galactocerebroside C; GQ1b, ganglioside quadrosialo 1b; GM1, ganglioside monosialo 1 (the numbers stand for the order of migration on thin-layer chromatography, and the lower-case letters stand for variations within basic structures); HMW, high-molecular-weight. Structures of *M. pneumoniae* adhesion proteins and host glycolipids are adapted from [33] and [34], respectively.

Interestingly, a promising diagnostic marker for *M. pneumoniae* encephalitis has recently emerged from a few case studies. In one study, intrathecal synthesis of antibodies to *M. pneumoniae* was reported in 14 cases of *M. pneumoniae* encephalitis (74%) [28]. The intrathecal production of antibodies is generally considered a highly specific marker for infection of the CNS [22]. All cases that underwent PCR testing (93%) indeed had a positive PCR targeting *M. pneumoniae* in the CSF [28] even though it has been recently demonstrated that intrathecal antibody responses to *M. pneumoniae* but not bacterial DNA can be present at the onset of clinical encephalitis [29]. In another study, it was reported that intrathecal antibodies to *M. pneumoniae* were found to cross-react with galactocerebroside C (GalC) in eight out of 21 (38%) of *M. pneumoniae* encephalitis cases [30]. All eight cases showed a negative PCR targeting *M. pneumoniae* in CSF. The cross-reactivity in these cases is likely induced by molecular mimicry between bacterial glycolipids and host myelin glycolipids, including GalC and gangliosides (Figure 2; figure references: [31]–[34]). Cross-reactive, anti-GalC antibodies have previously been detected in patients with Guillain-Barré syndrome (GBS) who suffered from a preceding *M. pneumoniae* infection [32], [35]–[38]. GBS is a typical postinfectious immune-mediated peripheral neuropathy [39]. In GBS, cross-reactive antibodies cause complement activation and formation of a membrane attack complex at the peripheral nerves, resulting in neuromuscular paralysis. Anti-GalC antibodies have been associated with demyelination in patients with GBS [35], [38]. Moreover, these anti-GalC antibodies cause neuropathy in rabbits that are immunized with GalC [40]. Such antibodies may also be involved in demyelination of central nerve cells in *M. pneumoniae* encephalitis, as a significant correlation was found between the presence of anti-GalC antibodies in the CSF and demyelination (*p* = 0.026) [30].

Anti-GalC antibodies have not only been detected in CSF but also in the serum of *M. pneumoniae* encephalitis patients [30], [36], [41]–[43], including rates from 13% (2/15) [30] to 100% (3/3) [41], respectively. It is possible that during inflammation the blood-brain barrier (BBB) can become permeable, which would thereby enable antibodies to cross the BBB and cause disease. As a consequence, the cross-reactive antibodies in the CSF of *M. pneumoniae* encephalitis patients do not necessarily have to be produced intrathecally (Figure 1).

*M. pneumoniae* infections may also be followed by the production of antibodies to gangliosides, both in patients with GBS and in those with encephalitis. In *M. pneumoniae* encephalitis, such antibodies were directed against GQ1b [44], [45] or GM1 [46] (Figure 2). Interestingly, anti-GQ1b antibodies are associated with a distinct and severe encephalitis variant, referred to as Bickerstaff brain stem encephalitis [47].

In conclusion, while PCR and serology may be of limited value in the diagnosis of *M. pneumoniae* encephalitis, the detection of intrathecal antibodies to *M. pneumoniae*, including cross-reactive antibodies against GalC and gangliosides, may be regarded as a promising new diagnostic tool.

The routine diagnostic workup of *M. pneumoniae* encephalitis should therefore aim for the detection of *M. pneumoniae* antibodies in both CSF and serum, in addition to *M. pneumoniae* PCR in CSF. Intrathecal antibodies can be detected by widely accessible enzyme immunoassays (EIAs) or immunoblotting (Table 1), while intrathecal antibody synthesis can be established either by calculation of an antibody index [22] or through parallel immunoblotting of simultaneously collected CSF and serum samples [48], [49]. Antiganglioside antibodies can be detected routinely by some specialized laboratories, but their detection together with cross-reactive antibodies against GalC primarily serve scientific purposes and may help to clarify *M. pneumoniae* antibodies' immune target(s). Furthermore, their hypothesized role in the pathogenesis might provide a basis for immunomodulatory treatment in *M. pneumoniae* encephalitis.
